# Relevance of 2′-O-Methylation and Pseudouridylation for the Malignant Melanoma

**DOI:** 10.3390/cancers13051167

**Published:** 2021-03-09

**Authors:** Simon Jasinski-Bergner, Juliane Blümke, Claudia Wickenhauser, Barbara Seliger

**Affiliations:** 1 Institute for Medical Immunology, Faculty of Medicine, Martin Luther University Halle-Wittenberg, Magdeburger Straße 2, 06108 Halle (Saale), Germany; juliane.bluemke@gmail.com (J.B.); Barbara.seliger@uk-halle.de (B.S.); 2 Institute for Pathology, Faculty of Medicine, Martin Luther University Halle-Wittenberg, 06108 Halle (Saale), Germany; claudia.wickenhauser@uk-halle.de; 3 Fraunhofer Institute for Cell Therapy and Immunology, 04103 Leipzig, Germany

**Keywords:** snoRNA, 2′-O-methylation, pseudouridylation, microRNA, malignant melanoma

## Abstract

**Simple Summary:**

This study investigates the expression, the histological localization, and the influence of the factors involved in 2′-O-methylation and pseudouridylation on prognostic relevant markers, proliferation markers, overall survival, molecular immune surveillance and evasion mechanisms within the malignant melanoma. Statistically significant positive correlations to the expression of markers involved in cell proliferation were observed. The upregulation of the RNA modifying factors was of prognostic relevance in this tumor disease with a negative impact on the overall survival of melanoma patients. Furthermore, the factors involved in 2′-O-methylation and pseudouridylation were statistically significant negative correlated to the expression of human leukocyte antigen class I genes as well as of components of the antigen processing machinery.

**Abstract:**

The two RNA modifications 2′-O-methylation and pseudouridylation occur on several RNA species including ribosomal RNAs leading to an increased translation as well as cell proliferation associated with distinct functions. Using malignant melanoma (MM) as a model system the proteins mediating these RNA modifications were for the first time analyzed by different bioinformatics tools and public available databases regarding their expression and histological localization. Next to this, the impact of these RNA-modifying factors on prognostic relevant processes and marker genes of malignant melanoma was investigated and correlated to immune surveillance and evasion strategies. The RNA modifying factors exerted statistically significant positive correlations to the expression of genes involved in cell proliferation and were statistically significant negative correlated to the expression of human leukocyte antigen class I genes as well as of components of the antigen processing machinery in malignant melanoma. Upregulation of the RNA modifying proteins was of prognostic relevance in this tumor disease with a negative impact on the overall survival of melanoma patients. Furthermore, the expression of known oncogenic miRs, which are induced in malignant melanoma, directly correlated to the expression of factors involved in these two RNA modifications.

## 1. Introduction

Malignant melanoma (MM) refers to a neoplasm of melanocytes comprising of neural crest-derived cells located in particular in the stratum basal of the skin’s epidermis and in the uvea of the eye. In contrast to keratinocytes, melanocytes are not linked to the basal lamina by hemidesmosomes and do not have desmosomes to neighboring keratinocytes. Instead, melanocytes as well as adjacent keratinocytes express, e.g., E-cadherin on their cell surface [1], which is an important factor contributing to the cell migration and invasion of melanoma cells after malignant transformation of melanocytes. Due to this migratory ability, growth characteristics as well as other properties including resistance to radiation and certain chemotherapeutics [2], MM represents the deadliest type of skin cancer with increasing incidences [3]. UV light exposition leading to DNA damages and oxidative stress in melanocytes [4], nevus number, pigmentation characteristics as well as genetic mutations are known predispositions for the processes leading to malignant transformation [5]. Concerning the hereditary predisposition, it is known that between 5–10% of melanoma cases are familial [6] and mainly induced by germ line mutations in tumor suppressor genes involved in proliferation control and DNA repair of cells.

Benign nevi defined as growth arrested, clonal neoplasms of melanocytes initiated by well-defined oncogenic mutations in the mitogen-activated protein kinase pathway, such as the BRAFV600E-activating mutation [7], may develop dysplastic features and are classified as MM precursor lesions.

An essential impact on the cell proliferation per se and exemplarily referred to MM is confined by ribosomes. An average mammalian cell has between 5 and 10 million ribosomes, which are large multi-subunit ribonucleoprotein complexes and are required for protein synthesis. Therefore, the formation of ribosomes and their linked translational efficiency (TE) directly influence cell proliferation, metabolism and vitality—parameters that are modulated upon malignant transformation [8] and indicative for neoplastic progression [9]. Thus, an enhanced TE is directly proportional to the ribosomal density, since the number of ribosomes on a coding transcript is linked to the efficacy of translation [10,11,12]. Indeed, an increase of ribosomes has been described in various cancers suggesting that the number and modifications of ribosomes drive tumorigenesis [13].

The size of the nucleoli as major location of the ribosomal assembly represents one important cytomorphologic parameter for the determination of malignancy in melanocytic lesions and has prognostic relevance [14]. Interestingly, expression of MYC as well as mutations in the tumor suppressor genes TP53 and RB1 enhanced ribosomal biogenesis [14,15].

Next to processes of transcription, the splicing and assembly of the eukaryotic 80 S ribosomes RNA modifications including 2′-O-methylation as well as the pseudouridylation occur in nucleoli, which involve a class of non-coding small RNAs termed sno-RNAs. Both processes represent the most common modifications of rRNAs and are important steps for their maturation as well as stabilization. In mammalian cells, the 5.8S rRNA, the 18S and the 28S rRNA have in total > 100 2′-O-methylations and 95 pseudouridinylations [16,17]. In addition to the rRNAs, the class of spliceosomal snoRNAs contain both modifications underlining the influence of snoRNAs for the splicing process [18]. The altered splicing pattern after malignant transformation involve mRNAs of many cancer related and tumor biological important genes [19]. In addition, transfer RNA (tRNA) molecules, microRNAs (miRs) and messenger RNAs (mRNAs) can be modified by 2′-O-methylation [20].

**Figure 1:**
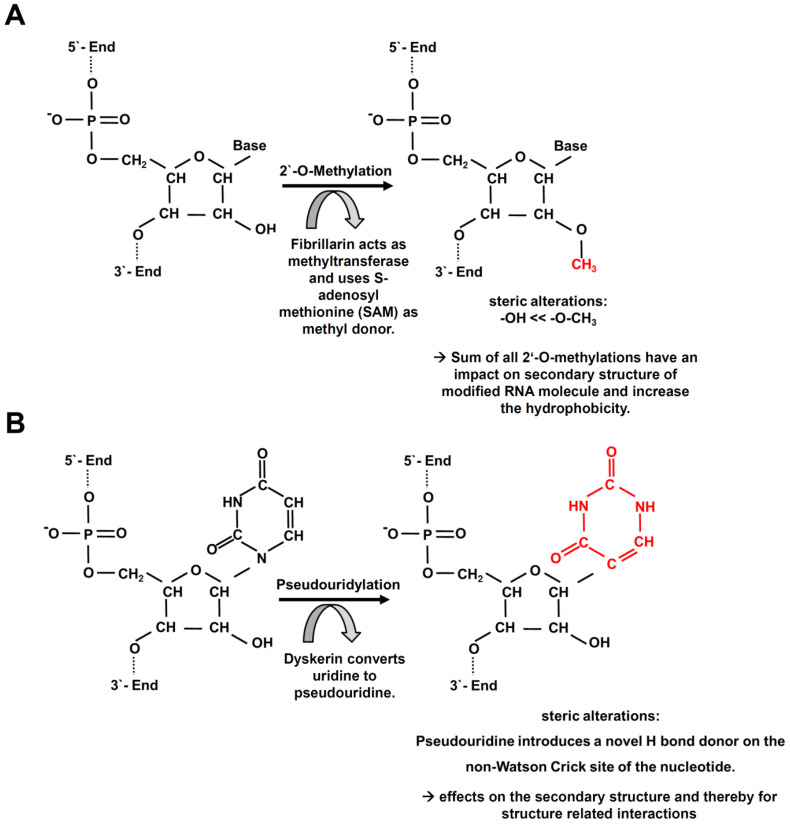
Molecular RNA modifications on RNAs involving snoRNAs. (**A**) 2′-O-methylation and (**B**) pseudouridylation.

For the 2′-O-methylation, a methyl residue is added to the ribose backbone, whereby fibrillarin (FBL) is the methyltransferase using S-adenosyl methionine (SAM) as a methyl donor (Figure 1A). FBL acts in a complex with nucleolar protein (NOP) 56, NOP58, 15.5K (SNU13) and the guide RNA [21]. The introduction of the methyl residue leads to steric alterations and increases the hydrophobicity thereby protecting RNA molecules from nucleolytic attacks [22]. The sum of all 2′-O-methylations within a RNA molecule can also affect the secondary structure and therefore possible interactions including RNA/RNA interactions, RNA-protein interactions [23,24] as well as mRNA splicing, stability and translation [25].

Many sites of the pseudouridylation are evolutionarily conserved [26,27]. In humans, the uridine is converted to pseudouridine in rRNA, sno/scaRNA and snRNA by dyskerin (DKC1), which is a component of a complex consisting of one H/ACA snoRNA and four core proteins, namely GAR1 ribonucleoprotein (GAR1), NHP2 ribonucleoprotein (NHP2), NOP10 ribonucleoprotein (NOP10) and DKC1 itself [28]. The substitution of uridine with pseudouridine introduces a novel H bond donor on the non-Watson Crick site of the nucleotide affecting the secondary structure and consequently structure related interactions [29] (Figure 1B). Pseudouridylation is involved in many important processes of gene expression including spliceosomal small nuclear ribonucleoprotein (snRNP) biogenesis, efficiency of pre-mRNA splicing and translation fidelity [30]. Another human pseudouridine synthase is PUS10 involved in the miR maturation, which results after its depletion in a reduced expression of mature miRs [31]. Furthermore, the 2′-O-methylation and the pseudouridylation have also an impact on the binding of RNA-binding proteins (RBPs) and are required for an appropriate mRNA splicing [25,30], which is altered in various tumor entities including MM [32,33,34]. In this study, the expression of key molecules involved in 2′-O-methylation and pseudouridylation is addressed in more detail using MM as a model.

## 2. Results

### 2.1. Expression Pattern and Localization of RNA-Modifying Proteins in the Skin

**Figure 2:**
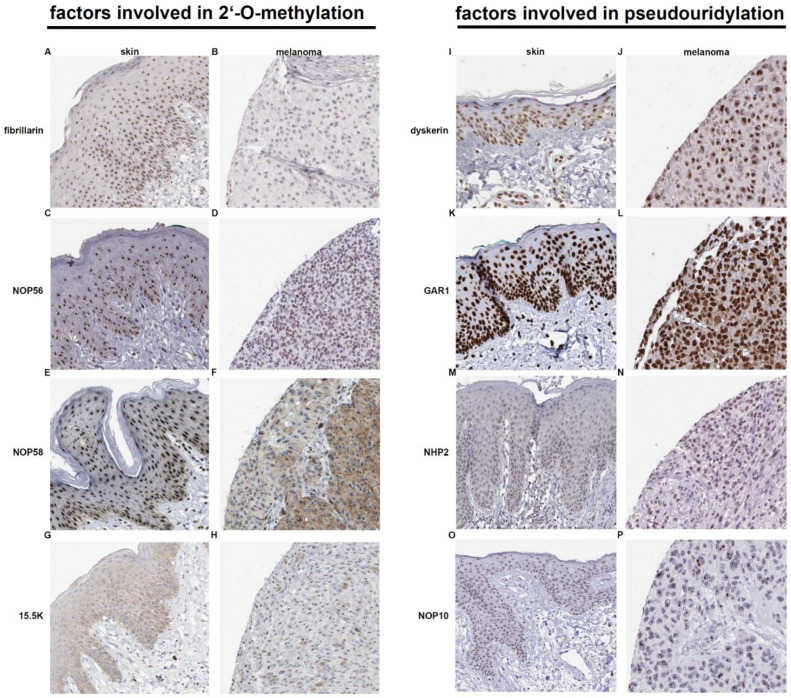
Representative immunohistochemical stainings of selected factors involved in 2′-O-methylation and pseudouridylation in human skin and melanoma specimen.

According to the information provided by The Human Protein Atlas, the protein expression involved in the pseudouridylation including DKC1, GAR1, NHP2 and NOP10 were only localized in the nucleus, while the staining of the proteins involved in 2′-O-methylation, namely FBL, NOP56, NOP58 as well as 15.5K (SNU13) revealed a more heterogenic localization summarized in Table 1. FBL, NOP56 and NOP58 were located in the nucleus and with the exception of NOP56 strongly expressed in the epidermis and in the highly proliferation active cells of the stratum germinativum. In contrast, the staining of 15.5K revealed a cytoplasmic and membranous expression almost exclusively in cells of the epidermis layers of the skin (Table 1, Figure 2).

**Figure 3:**
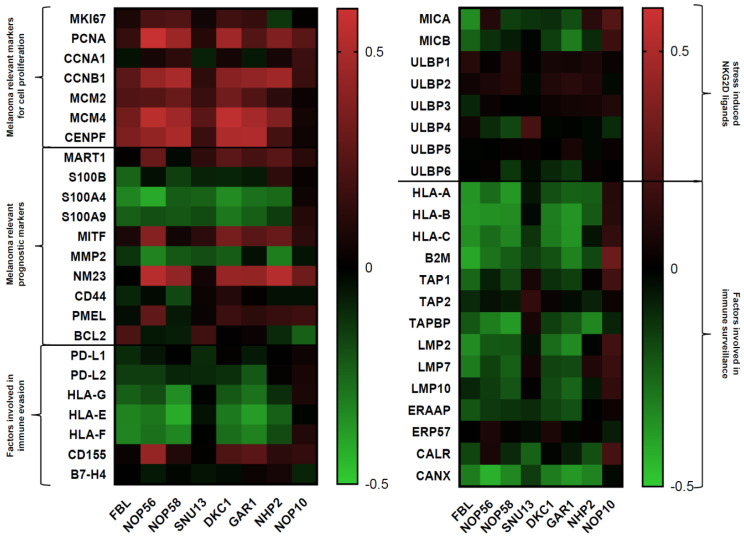
Summarizing visualization as a heatmap of the positive and negative correlations of factors involved in 2′-O-methylation and pseudouridylation with melanoma relevant markers of cell proliferation, prognostic markers, and genes involved in immune surveillance as well as immune evasion.

The correlations of factors involved in 2′-O-methylation and pseudouridylation with melanoma relevant proliferation markers, prognostic markers, and with genes responsible for immune surveillance as well as for immune evasion and positive as well as negative regulations were seen as indicated and visualized in the summarizing heatmap in Figure 3. The respective R and *p* values are listed within Table 2, Table 3 and Table 4. The results will be addressed in more details in the following paragraphs.

### 2.2. Correlation of RNA-Modifying Proteins with Tumor Cell Proliferation

To address whether the expression of factors involved in 2′-O-methylation and pseudouridylation correlates with the pathological increased proliferation rates in MM, the expression data of these RNA-modifying factors were analyzed in The Cancer Genome Atlas (TCGA) data sets consisting of 214 samples from MM patients provided by the R2 data base. The following proliferation markers known to play a role in MM were investigated: Ki67 (MKI67), PCNA, cyclin A (CCNA1), cyclin B (CCNB1), MCM2, MCM4, and mitosin (CENPF) [35,36,37,38].

As summarized in Table 2, positive correlations between both RNA modifying factors and the proliferation markers analyzed were found. The effect was the strongest for MCM4, which correlated statistically significant to all factors involved into 2′-O-methylation and pseudouridylation with the exception of NOP10. In addition, PCNA, cyclin B and mitosin showed strong correlations, while the expression of CCNA1 did not correlate statistically significant with any of these factors.

Based on the strong positive correlation between the factors involved in 2′-O-methylation and pseudouridylation with most of the clinical relevant proliferation markers, a possible correlation between these factors and prognostic marker genes relevant for MM was evaluated such as melanoma antigen recognized by T cells (MART)1, S100 calcium binding protein B (S100B), S100A4, S100A9, melanocyte inducing transcription factor (MITF), matrix metallopeptidase 2 (MMP2), nucleoside diphosphate kinase 1 (NM23), cluster of differentiation (CD) 44, premelanosome protein (PMEL), and BCL2 apoptosis regulator (BCL2) using the same TCGA data sets [39,40,41,42,43] (Table 3). Next to these marker genes the invasion depth termed Breslow’s depth is of prognostic value. The average Breslow score of the 214 MM patients analyzed was 2.5 mm. All other clinical parameters of this MM patient cohort (*n* = 214) of the analyzed microarray data can be obtained from the original literature Jönsson et al., 2015 [44].

The two markers MART1 and MITF, but also NM23 showed statistically significant positive correlations to the expression of RNA modifying proteins. In contrast, the S100 family members S100B, S100A4, S100A9 and MMP2 exhibiting a prognostic potential in MM exerted a statistically significant negative correlation, whereas CD44 did not show any correlation and PMEL as well as BCL2 were only weakly positivey correlated.

**Figure 4:**
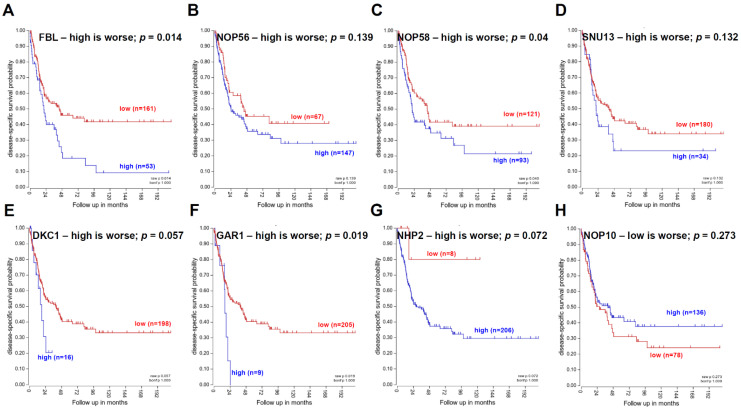
Impact of factors involved in 2′-O-methylation and pseudouridylation on the overall survival in MM. (**A**–**H**): Kaplan–Meier Plots were generated with TCGA data of 214 melanoma specimens provided by the R2 database (https://hgserver1.amc.nl/ (accessed on 12 February 2021)).

To address whether the factors involved in 2′-O-methylation and pseudouridylation are of central importance for prognosis in MM, the same TCGA data set was used for the generation of Kaplan–Meier plots. As shown in Figure 4A–H, a direct correlation of the expression level of these factors with the overall survival (OS) of MM patients was detected and statistically significant for FBL, NOP58, and GAR1. In contrast, low NOP10 expression levels predicted a statistically non-significant correlation with a worse patients’ outcome (Figure 4). However, it is noteworthy that the use of TCGA data cannot replace extensive protein based analyses of ex vivo MM specimens.

### 2.3. Correlation of the Expression of RNA Modifying Factors with Immune Modulatory Genes

Due to the increased implementation of immunotherapies for the treatment of hematopoietic and solid tumors, it was analyzed whether the factors involved in 2′-O-methylation and pseudouridylation directly or indirectly affect transcripts participating in the immune surveillance of tumor cells. Therefore, their expression patterns were correlated to molecules involved in the immune recognition/evasion of malignant and/or virus transformed cells from immune effector cells. Using the same melanoma data set, an impressive statistically significant negative correlation between immunological relevant molecules, in particular the human leucocyte antigen (HLA) class Ia and Ib and components of the HLA class I antigen processing machinery (APM), with the factors involved in 2′-O-methylation and pseudouridylation was detected (Table 4). This might also explain the negative correlation of these factors with the OS of MM patients, since their impaired expression was associated with a reduced anti-tumoral immune cell response [45] (Figure 4). The divergent statistically significant positive correlation pattern of NOP10 to molecules involved in immune surveillance was also associated with a statistically significant positive correlation to the OS in MM patients (Figure 4H). However, further studies are needed to investigate whether the statistically significant negative correlated immunological relevant genes are directly negatively regulated upon a reduced activity/expression of the factors involved 2′-O-methylation and pseudouridylation.

### 2.4. Correlation of miR Expression with RNA-Modifying Factors

The processes of 2′-O-methylation and pseudouridylation also occur in other RNA species with a strong impact to diverse molecular processes of malignant transformation and thus are of clinical relevance. These other RNA species include microRNAs (miRs), which are non-coding single stranded RNAs with an approximately length of ~22 nt [46], binding specifically and preferentially, but not exclusively, within the 3′- untranslated region (UTR) to their target mRNAs sequence [40,47] thereby causing a translational repression and mRNA decay [48].

Some miRs can be classified into oncogenic, tumor suppressive and/or immune modulatory miRs [48]. In addition, human viruses including herpes viruses encode for viral miRs, which also affect cancer related cellular processes [49].

Several oncogenic miRs have been reported to be frequently overexpressed in MM. Fattore and co-authors (2017) even grouped the most representative deregulated miRs in melanoma with regard to the different steps of tumor progression. These potentially oncogenic miRs with putative diagnostic and/or prognostic values include miR-9, miR-10a, miR-10b, miR-17-5p, miR-18a, miR-21, miR-26b, miR-92a, miR-221, miR-222, miR-126, miR-145, miR146, miR-182, miR-514, miR-520d and miR-527 [50].

The pseudouridylation of different human RNA species is mediated by different pseudouridine synthases, such as pseudouridine synthase (PUS) 1, TruB pseudouridine synthase 2 (TRUB2), TRUB1, PUS3, PUS4, RNA pseudouridine synthase D3 (RPUSD3), RPUSD4, PUS7, pseudouridine synthase 7 like (PUS7L), PUS10 and DKC1 [28]. From these candidates, TRUB1 has been recently reported to modulate the stability of hsa-let-7 [51]. However, it is noteworthy that the actual list of enzymes involved in 2′-O-methylation and pseudouridylation of miRs might be incomplete.

Thus far, only little is known, which of these enzymes are able to modify miRs and which alterations in the respective miR-mediated functions and/or miR stability are caused by such modifications.

To determine whether the expression of known oncogenic miRs in MM might be correlated with the expression of enzymes involved into 2′-O-methylation and pseudouridylation, the TCGA data of 214 human MM samples were further analyzed for miR expression. As shown in Table 5, there existed no evidence for a global impact of the induction of oncogenic miRs, while miR-21 was statistically significant negatively correlated to the expression of the investigated enzymes in MM.

Analysis of the TCGA data from 214 human melanoma tumors provided by the R2 database (https://hgserver1.amc.nl/ (accessed on 12 February 2021)) for correlation with enzymes known to be involved in miR 2′-O-methylation and pseudouridylation with the amount of increased and/or stabilized oncogenic miRs reported to be induced in MM.

## 3. Discussion

Recently, the aberrant expression pattern of DKC1, NHP2, and NOP10 in several cancer entities has been reviewed [52]. Due to their biological functions, the localization of these factors was almost completely limited to the nucleus, since 2′-O-methylation and pseudouridylation occur within the nucleoli. Furthermore, an increase within the highly proliferational active cells of the epidermal stratum germinativum was demonstrated, which reflects the involvement and increased number of ribosomes during proliferation. This was further underlined by statistically significant positive correlations to proliferation markers suggesting that some factors involved in 2′-O-methylation and pseudouridylation might also be suitable markers for proliferation themselves.

However, this study did not answer whether in the case of positive correlations between the factors involved in 2′-O-methylation and pseudouridylation and, e.g., proliferation markers these positive correlations are an indirect result due to an enhanced proliferation per se mediated by increased translational efficacy caused by modifications within ribosomal RNAs, or whether the mRNAs of the positively correlated proliferation markers are directly modified by these factors or by both mechanisms in parallel.

The positive correlations described in this study are based upon TCGA data, not taken posttranscriptional mechanisms of gene regulation into account. Therefore, in depth protein-based studies applying human melanoma tissue specimens are required to proof this hypothesis.

In regard to the observed statistically significant negative and coordinated correlations to molecules involved in immune surveillance/evasion it is noteworthy that (i) HLA Ia and Ib genes, HLA class related MICs and major APM components are located within the major histocompatibility complex (MHC) locus on chromosome 6p21 and that (ii) the adaptive immunity phylogenetically occurs with the jawed vertebrates [53] and, e.g., HLA class Ib genes are evolutionary even younger, which might have an impact on the observed negative correlations.

Due to the limitation of transcriptome based microarray data sets it is necessary to underline that also indirect effects may cause such down regulations. The increased proliferation itself might decrease the antigen processing and presentation efficacy in melanoma cells. In turn, the impaired antigen processing and presentation is a frequent immune evasion strategy of tumors [54], which might be a prerequisite for an increased proliferation rate of tumor cells. Thus far, the sequence of these events in MM has not been studied.

**Figure 5:**
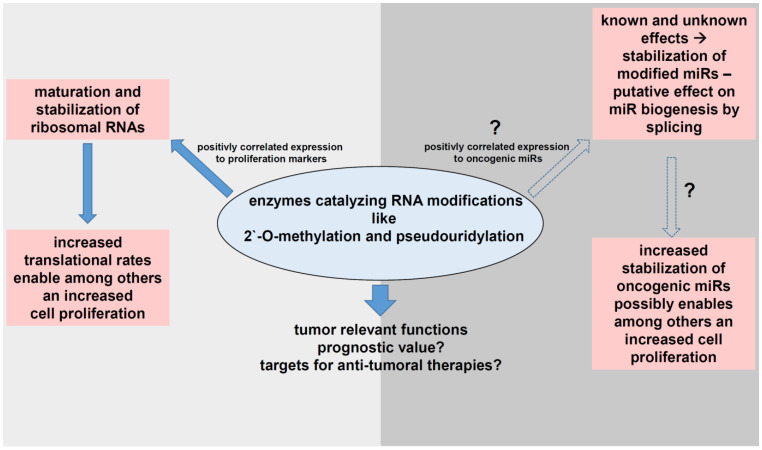
Impact of the RNA modifications analyzed on tumor relevant processes.

RNA modifications on miRs affecting their stability or decay might also indirectly contribute to the regulation of cancer related processes, even independently of the translational processes linked to the number of ribosomes. This opens a new regulatory dimension for RNA modifying enzymes (Figure 5), which has to be explored in more detail.

Thus far, it is controversially discussed whether mature mammalian miRs contain 2′-O-methylations as reported for plants and *Drosophila* [55]. In *Drosophila*, 2′-O-methylation of miRs occurred age dependent and its inhibition resulted in an accelerated neurodegeneration and shorter life span [56]. Despite discrepancies regarding the missing 2′-O-methylation in mammalian miRs, the human miR-21-5p has been shown to contain a 3′-terminal 2′-O-methylation, which enhances the stability of this oncogenic miR in lung cancer patients. Interestingly, HENMT1 was identified to act as methyltransferase [57].

Indirect effects concerning the positive or negative correlations of factors involved in 2′-O-methylation and pseudouridylation with miRs might occur. Several miRs are processed from introns after the splicing, while for the process of splicing the snoRNAs as huge group of the snRNAs in involved. These snoRNAs harbor themselves 2′-O-methylations and pseudouridylations contributing to the stability and functionality of the snoRNAs.

In this study, the impact and relevance of factors involved in 2′-O-methylation and pseudouridylation of different RNA species related to the processes of tumor formation and progression were summarized in MM as a tumor model. Using different bioinformatics tools statistically significant positive correlations between proliferation and prognostic marker genes relevant for the MM with the factors involved into 2′-O-methylation and pseudouridylation were described for the first time.

The impact of these molecules on other RNA species including miRs has recently been investigated, but needs to be further studied in detail. This will lead to the identification of proteins involved in such miR modifications, which might have an impact on the function of such modified miRs. However, the existence of miR 2′-O-methylation and pseudouridylation in general must not necessarily involve tumor relevant miRs and processes.

## 4. Materials and Methods

### Immunohistochemistry

The frequency and localization of protein levels involved in the two RNA modification processes, namely FBL, NOP56, NOP58, 15.5K (SNU13), DKC1, GAR1, NHP2, and NOP10, were analyzed by evaluation of immunohistochemistry data of healthy normal skin sections and melanoma specimen provided by the free available online data base. The Human Protein Atlas [58,59,60].

Bioinformatic analyses of gene expression data and correlation with patients’ overall survival.

The factors involved in RNA 2′-O-methylation and pseudouridylation were correlated with genes encoding for above mentioned ribosomal RNAs carrying multiple of such modifications. Unfortunately, the applied Microarrays (Illumina Human HT-12V4.0 Chips) of the 214 MM patients published by Jönsson and co-authors 2015 [44] contained only probes against the human RN5S9 transcript encoding the 5S ribosomal RNA. The factors showed a positive correlation to the RN5S9, which was statistically significant for NOP56, NOP58 and NHP2.

The correlation coefficients reflecting R values and the respective *p* values were calculated by the R2 database (https://hgserver1.amc.nl/ (accessed on 12 February 2021)). For visualization these R values were presented in a heatmap generated by GraphPad Prism 8 (GraphPad Software, San Diego, CA, USA).

Correlation of the expression pattern of genes involved into 2′-O-methylation and pseudouridylation with preselected genes involved in cell proliferation, prognosis and immune recognition/evasion in MM is based upon TCGA data of 214 melanoma specimens provided by the R2 database (https://hgserver1.amc.nl/ (accessed on 12 February 2021)). Statistically significant positive or negative correlations (*p* < 0.05) are highlighted in red or green. The same TCGA data set was applied for the generation of the respective Kaplan–Meier Plots.

## 5. Conclusions

Thus, a link between 2′-O-methylation and pseudouridylation to cell proliferation, host immunity and oncogenic miRs exists in MM suggesting that both RNA modifications and factors involved in this process represent suitable targets for tumor therapy and putative novel prognostic markers.

## Figures and Tables

**Table 1:** Localization of the proteins involved into 2′-O-methylation and pseudouridylation in skin and MM specimen based upon the information provided by The Human Protein Atlas.

RNA Modification	Factor	Localization within Skin	Localization within MM
factors involved into 2′-O-methylation	FBL	nuclear, extra strong within the cells of the epidermis especially in the stratum germinativum	nuclear
	NOP56	nuclear	nuclear
	NOP58	nuclear strongest expression within the cells of the epidermal layers, especially within the stratum germinativum	cytoplasmic and nuclear
	15.5K (SNU13)	cytoplasmic and membranous restricted to the cells of the epidermis	nuclear
factors involved into pseudouridylation	DKC1	nuclear	nuclear
	GAR1	nuclear	nuclear
	NHP2	nuclear	nuclear
	NHP10	nuclear	nuclear

**Table 2:** Correlation of the factors involved in 2′-O-methylation and pseudouridylation with known proliferation markers in MM.

Correlated Expression	FBL	NOP56	NOP58	SNU13	DKC1	GAR1	NHP2	NOP10
MKI67	R = 0.081	R = 0.216	R = 0.235	R = 0.050	R = 0.185	R = 0.154	R = −0.133	R = 0.011
	*p* = 0.241	*p* = 1.47 × 10^−3^	*p* = 5.22 × 10^−4^	*p* = 0.468	*p* = 6.50 × 10^−3^	*p* = 0.024	*p* = 0.053	*p* = 0.874
PCNA	R = 0.152	R = 0.581	R = 0.448	R = 0.106	R = 0.463	R = 0.238	R = 0.372	R = 0.258
	*p* = 0.026	*p* = 9.59 × 10^−21^	*p* = 5.58 × 10^−12^	*p* = 0.122	*p* = 9.06 × 10^−13^	*p* = 4.55 × 10^−4^	*p* = 1.92 × 10^−8^	*p* = 1.36 × 10^−4^
CCNA1	R = −0.046	R = 0.065	R = 0.131	R = −0.079	R = 0.060	R = −0.063	R = 0.066	R = 0.172
	*p* = 0.499	*p* = 0.341	*p* = 0.056	*p* = 0.250	*p* = 0.383	*p* = 0.356	*p* = 0.339	*p* = 0.011
CCNB1	R = 0.265	R = 0.439	R = 0.492	R = 0.136	R = 0.398	R = 0.423	R = 0.463	R = 0.165
	*p* = 8.62 × 10^−5^	*p* = 1.78 × 10^−11^	*p* = 1.88 × 10^−14^	*p* = 0.047	*p* = 1.50 × 10^−9^	*p* = 1.10 × 10^−10^	*p* = 9.44 × 10^−13^	*p* = 0.015
MCM2	R = 0.236	R = 0.251	R = 0.302	R = 0.166	R = 0.325	R = 0.233	R = 0.123	R = 0.031
	*p* = 4.94 × 10^−4^	*p* = 2.07 × 10^−4^	*p* = 6.94 × 10^−6^	*p* = 0.015	*p* = 1.16 × 10^−6^	*p* = 5.80 × 10^−4^	*p* = 0.072	*p* = 0.656
MCM4	R = 0.334	R = 0.546	R = 0.466	R = 0.262	R = 0.554	R = 0.482	R = 0.377	R = 0.057
	*p* = 5.60 × 10^−7^	*p* = 4.78 × 10^−18^	*p* = 6.15 × 10^−13^	*p* = 1.04 × 10^−4^	*p* = 1.34 × 10^−18^	*p* = 7.93 × 10^−14^	*p* = 1.29 × 10^−8^	*p* = 0.403
CENPF	R = 0.363	R = 0.428	R = 0.495	R = 0.162	R = 0.509	R = 0.515	R = 0.198	R = 0.040
	*p* = 4.69 × 10^−8^	*p* = 6.01 × 10^−11^	*p* = 1.33 × 10^−14^	*p* = 0.018	*p* = 1.62 × 10^−15^	*p* = 6.59 × 10^−16^	*p* = 3.61 × 10^−3^	*p* = 0.556

Based on TCGA data of 214 human MM samples provided by the R2 database (https://hgserver1.amc.nl/ (accessed on 12 February 2021)), statistically significant positive correlations (*p* < 0.05) of proteins in 2′-O-methylation and pseudouridylation to proliferation markers of MM were found and highlighted in red. Background color: One group of factors belong to the brighter grey. The 2nd group to the darker grey.

**Table 3:** Correlation of the factors involved in 2′-O-methylation and pseudouridylation with known prognostic markers in MM.

Correlated Expression	FBL	NOP56	NOP58	SNU13	DKC1	GAR1	NHP2	NOP10
MART1	R = 0.009	R = 0.304	R = −0.024	R = 0.134	R = 0.265	R = 0.203	R = 0.259	R = 0.115
	*p* = 0.893	*p* = 6.12 × 10^−6^	*p* = 0.728	*p* = 0.050	*p* = 8.59 × 10^−5^	*p* = 2.92 × 10^−3^	*p* = 1.25 × 10^−4^	*p* = 0.094
S100B	R = −0.248	R = −0.038	R = −0.152	R = −0.085	R = −0.090	R = −0.067	R = 0.133	R = 0.021
	*p* = 2.53 × 10^−4^	*p* = 0.577	*p* = 0.026	*p* = 0.218	*p* = 0.191	*p* = 0.327	*p* = 0.053	*p* = 0.757
S100A4	R = −0.325	R = −0.414	R = −0.223	R = −0.240	R = −0.342	R = −0.274	R = −0.258	R = 0.042
	*p* = 1.20 × 10^−6^	*p* = 2.79 × 10^−10^	*p* = 1.01 × 10^−3^	*p* = 3.96 × 10^−4^	*p* = 2.98 × 10^−7^	*p* = 4.75 × 10^−5^	*p* = 1.33 × 10^−4^	*p* = 0.539
S100A9	R = −0.234	R = −0.192	R = −0.212	R = −0.185	R = −0.295	R = −0.234	R = −0.147	R = 0.104
	*p* = 5.57 × 10^−4^	*p* = 4.92 × 10^−3^	*p* = 1.81 × 10^−3^	*p* = 6.72 × 10^−3^	*p* = 1.13 × 10^−5^	*p* = 5.58 × 10^−4^	*p* = 0.031	*p* = 0.130
MITF	R = 0.076	R = 0.388	R = 0.050	R = 0.124	R = 0.345	R = 0.256	R = 0.304	R = 0.124
	*p* = 0.271	*p* = 4.05 × 10^−9^	*p* = 0.468	*p* = 0.070	*p* = 2.24 × 10^−7^	*p* = 1.51 × 10^−4^	*p* = 5.90 × 10^−6^	*p* = 0.070
MMP2	R = −0.129	R = −0.321	R = −0.271	R = −0.190	R = −0.226	R = −0.040	R = −0.309	R = −0.050
	*p* = 0.059	*p* = 1.67 × 10^−6^	*p* = 5.96 × 10^−5^	*p* = 5.20 × 10^−3^	*p* = 8.54 × 10^−4^	*p* = 0.561	*p* = 4.10 × 10^−6^	*p* = 0.465
NM23	R = −0.005	R = 0.534	R = 0.424	R = 0.053	R = 0.442	R = 0.425	R = 0.531	R = 0.321
	*p* = 0.944	*p* = 3.56 × 10^−17^	*p* = 9.77 × 10^−11^	*p* = 0.445	*p* = 1.21 × 10^−11^	*p* = 8.80 × 10^−11^	*p* = 5.60 × 10^−17^	*p* = 1.66 × 10^−6^
CD44	R = −0.091	R = −0.026	R = −0.174	R = 0.034	R = 0.101	R = 0.012	R = −0.040	R = −0.040
	*p* = 0.184	*p* = 0.705	*p* = 0.011	*p* = 0.618	*p* = 0.139	*p* = 0.863	*p* = 0.557	*p* = 0.556
PMEL	R = −0.033	R = 0.281	R = −0.061	R = 0.029	R = 0.171	R = 0.126	R = 0.160	R = 0.179
	*p* = 0.630	*p* = 2.96 × 10^−5^	*p* = 0.371	*p* = 0.670	*p* = 0.012	*p* = 0.066	*p* = 0.019	*p* = 8.58 × 10^−3^
BCL2	R = 0.217	R = −0.059	R = −0.072	R = 0.183	R = 0.008	R = 0.031	R = −0.101	R = −0.235
	*p* = 1.40 × 10^−3^	*p* = 0.392	*p* = 0.292	*p* = 7.21 × 10^−3^	*p* = 0.907	*p* = 0.652	*p* = 0.140	*p* = 5.15 × 10^−4^

Based on TCGA data of 214 MM samples provided by the R2 database (https://hgserver1.amc.nl/ (accessed on 12 February 2021)) statistically significant positive (red) or negative (green) correlations (*p* < 0.05) of proteins in 2′-O-methylation and pseudouridylation to prognostic markers of MM are highlighted. Background color: One group of factors belong to the brighter grey. The 2nd group to the darker grey.

**Table 4:** Correlation of the expression of factors involved in 2′-O-methylation and pseudouridylation with preselected molecules involved into immune recognition/evasion.

	Correlated Expression	FBL	NOP56	NOP58	SNU13	DKC1	GAR1	NHP2	NOP10
					(NHP2L1)				
molecules contributing to recognition by immune effector cells	MICA	R = −0.341	R = 0.100	R = −0.152	R = −0.128	R = −0.112	R = −0.189	R = 0.121	R = 0.245
		*p =* 3.10 × 10^−7^	*p* = 0.146	*p* = 0.026	*p* = 0.061	*p* = 0.103	*p* = 5.59 × 10^−3^	*p* = 0.079	*p * = 2.88 × 10^−4^
	MICB	R = −0.241	R = −0.119	R = −0.069	R = −0.014	R = −0.149	R = −0.304	R = −0.106	R = 0.175
		*p =* 3.72 × 10^−4^	*p* = 0.082	*p* = 0.314	*p* = 0.834	*p* = 0.029	*p* = 5.75 × 10^−6^	*p* = 0.121	*p* = 0.010
	ULBP1	R = 0.109	R = 0.020	R = 0.109	R = 0.011	R = 0.067	R = 0.056	R = 0.077	R = 0.029
		*p* = 0.112	*p* = 0.767	*p* = 0.112	*p* = 0.877	*p* = 0.328	*p* = 0.412	*p* = 0.264	*p* = 0.677
	ULBP2	R = 0.047	R = 0.078	R = 0.107	R = −0.020	R = 0.096	R = 0.120	R = 0.093	R = −0.023
		*p* = 0.496	*p* = 0.255	*p* = 0.117	*p* = 0.766	*p* = 0.162	*p* = 0.080	*p* = 0.176	*p* = 0.742
	ULBP3	R = −0.091	R = 0.028	R = 0.005	R = −0.008	R = 0.036	R = 0.058	R = 0.060	R = 0.098
		*p* = 0.185	*p* = 0.688	*p* = 0.947	*p* = 0.912	*p* = 0.603	*p* = 0.398	*p* = 0.380	*p* = 0.154
	ULBP4	R = 0.048	R = −0.104	R = −0.171	R = 0.202	R = −0.015	R = −0.008	R = −0.024	R = −0.107
		*p* = 0.488	*p* = 0.129	*p* = 0.012	*p* = 2.93 × 10^−3^	*p* = 0.825	*p* = 0.906	*p* = 0.729	*p* = 0.117
	ULBP5	R = −0.011	R = −0.007	R = 0.018	R = 0.022	R = −0.001	R = 0.069	R = −0.022	R = 0.024
		*p* = 0.867	*p* = 0.915	*p* = 0.791	*p* = 0.744	*p* = 1.000	*p* = 0.313	*p* = 0.752	*p* = 0.730
	ULBP6	R = −0.005	R = 0.020	R = −0.132	R = −0.029	R = −0.093	R = −0.138	R = 0.025	R = −0.004
		*p* = 0.940	*p* = 0.772	*p* = 0.055	*p* = 0.674	*p* = 0.175	*p* = 0.044	*p* = 0.715	*p* = 0.951
	HLA-A	R = −0.364	R = −0.261	R = −0.370	R = −0.054	R = −0.195	R = −0.238	R = −0.229	R = 0.110
		*p =* 4.16 × 10^−8^	*p* = 1.13 × 10^−4^	*p* = 2.47 × 10^−8^	*p* = 0.431	*p* = 4.12 × 10^−3^	*p* = 4.55 × 10^−4^	*p * = 7.55 × 10^−4^	*p* = 0.109
	HLA-B	R = −0.369	R = −0.353	R = −0.342	R = −0.024	R = −0.305	R = −0.365	R = −0.218	R = 0.114
		*p =* 2.66 × 10^−8^	*p* = 1.16 × 10^−7^	*p* = 2.81 × 10^−7^	*p* = 0.723	*p* = 5.60 × 10^−6^	*p* = 3.92 × 10^−8^	*p * = 1.30 × 10^−3^	*p* = 0.096
	HLA-C	R = −0.351	R = −0.264	R = −0.323	R = −0.126	R = −0.298	R = −0.360	R = −0.048	R = 0.145
		*p =* 1.29 × 10^−7^	*p* = 9.52 × 10^−5^	*p* = 1.35 × 10^−6^	*p* = 0.066	*p* = 9.08 × 10^−6^	*p* = 5.96 × 10^−8^	*p* = 0.481	*p* = 0.034
	B2M	R = −0.407	R = −0.281	R = −0.224	R = −0.150	R = −0.198	R = −0.318	R = −0.178	R = 0.314
		*p =* 6.26 × 10^−10^	*p* = 2.97 × 10^−5^	*p* = 9.60 × 10^−4^	*p* = 0.028	*p* = 3.67 × 10^−3^	*p* = 2.04 × 10^−6^	*p * = 9.12 × 10^−3^	*p * = 2.73 × 10^−6^
	TAP1	R = −0.251	R = −0.074	R = −0.176	R = 0.067	R = −0.114	R = −0.154	R = 0.018	R = 0.189
		*p =* 2.05 × 10^−4^	*p* = 0.278	*p* = 9.93 × 10^−3^	*p* = 0.331	*p* = 0.097	*p* = 0.024	*p* = 0.788	*p * = 5.58 × 10^−3^
	TAP2	R = −0.102	R = −0.042	R = −0.064	R = 0.144	R = 0.027	R = −0.031	R = −0.081	R = 0.034
		*p* = 0.137	*p* = 0.538	*p* = 0.355	*p* = 0.035	*p* = 0.695	*p* = 0.655	*p* = 0.241	*p* = 0.624
	TAPBP	R = −0.210	R = −0.309	R = −0.374	R = 0.070	R = −0.156	R = −0.219	R = −0.328	R = −0.088
		*p =* 1.99 × 10^−3^	*p* = 3.94 × 10^−6^	*p* = 1.70 × 10^−8^	*p* = 0.311	*p* = 0.022	*p* = 1.25 × 10^−3^	*p * = 9.18 × 10^−7^	*p* = 0.19
	LMP2	R = −0.342	R = −0.214	R = −0.209	R = −0.038	R = −0.262	R = −0.338	R = −0.014	R = 0.190
		*p =* 2.96 × 10^−7^	*p* = 1.60 × 10^−3^	*p* = 2.07 × 10^−3^	*p* = 0.577	*p* = 1.03 × 10^−4^	*p* = 4.19 × 10^−7^	*p* = 0.833	*p =* 5.41 × 10^−3^
	LMP7	R = −0.299	R = −0.159	R = −0.233	R = 0.055	R = −0.164	R = −0.180	R = 0.092	R = 0.165
		*p =* 8.42 × 10^−6^	*p* = 0.020	*p* = 5.83 × 10^−4^	*p* = 0.420	*p* = 0.016	*p* = 8.24 × 10^−3^	*p* = 0.179	*p* = 0.015
	LMP10	R = −0.092	R = −0.148	R = −0.205	R = 0.008	R = −0.180	R = −0.245	R = −0.057	R = 0.138
		*p* = 0.180	*p* = 0.030	*p* = 2.55 × 10^−3^	*p* = 0.909	*p* = 8.33 × 10^−3^	*p* = 2.99 × 10^−4^	*p* = 0.406	*p* = 0.044
	ERAAP	R = −0.208	R = −0.141	R = −0.122	R = −0.104	R = −0.161	R = −0.197	R = −0.006	R = 0.036
		*p =* 2.24 × 10^−3^	*p* = 0.040	*p* = 0.075	*p* = 0.131	*p* = 0.019	*p* = 3.73 × 10^−3^	*p* = 0.932	*p* = 0.602
	ERP57	R = 0.004	R = 0.077	R = 0.014	R = −0.031	R = 0.081	R = −0.017	R = 0.012	R = −0.073
		*p* = 0.951	*p* = 0.262	*p* = 0.841	*p* = 0.648	*p* = 0.239	*p* = 0.804	*p* = 0.859	*p* = 0.285
	CALR	R = −0.169	R = 0.073	R = −0.102	R = −0.241	R = −0.010	R = −0.072	R = −0.191	R = 0.204
		*p* = 0.013	*p* = 0.288	*p* = 0.138	*p* = 3.64 × 10^−4^	*p* = 0.888	*p* = 0.296	*p * = 5.11 × 10^−3^	*p * = 2.74 × 10^−3^
	CANX	R = −0.303	R = −0.430	R = −0.332	R = −0.147	R = −0.300	R = −0.370	R = −0.320	R = −0.022
		*p =* 6.34 × 10^−6^	*p* = 4.64 × 10^−11^	*p* = 6.62 × 10^−7^	*p* = 0.031	*p* = 8.14 × 10^−6^	*p* = 2.48 × 10^−8^	*p * = 1.79 × 10^−6^	*p* = 0.744
									
molecules contributing to immune evasion	PD-L1 (B7-H1)	R = −0.107	R = −0.051	R = −0.004	R = −0.103	R = 0.010	R = −0.062	R = 0.003	R = 0.041
		*p* = 0.117	*p* = 0.460	*p* = 0.954	*p* = 0.132	*p* = 0.888	*p* = 0.363	*p* = 0.966	*p* = 0.548
	PD-L2 (PDCD1LG2)	R = −0.151	R = −0.150	R = −0.092	R = −0.096	R = −0.114	R = −0.215	R = 0.014	R = 0.068
		*p* = 0.027	*p* = 0.028	*p* = 0.182	*p* = 0.163	*p* = 0.097	*p* = 1.59 × 10^−3^	*p* = 0.838	*p* = 0.319
	HLA-G	R = −0.230	R = −0.190	R = −0.345	R = −0.008	R = −0.217	R = −0.280	R = −0.111	R = 0.081
		*p =* 6.90 × 10^−4^	*p* = 5.32 × 10^−3^	*p* = 2.20 × 10^−7^	*p* = 0.905	*p* = 1.39 × 10^−3^	*p* = 3.19 × 10^−5^	*p* = 0.104	*p* = 0.236
	HLA-E	R = −0.321	R = −0.290	R = −0.418	R = −0.045	R = −0.296	R = −0.380	R = −0.236	R = −0.013
		*p =* 1.59 × 10^−6^	*p* = 1.65 × 10^−5^	*p* = 1.95 × 10^−10^	*p* = 0.509	*p* = 1.06 × 10^−5^	*p* = 9.50 × 10^−9^	*p * = 4.92 × 10^−4^	*p* = 0.845
	HLA-F	R = −0.323	R = −0.274	R = −0.329	R = −0.005	R = −0.267	R = −0.318	R = −0.182	R = 0.098
		*p =* 1.41 × 10^−6^	*p* = 4.71 × 10^−5^	*p* = 8.52 × 10^−7^	*p* = 0.944	*p* = 7.79 × 10^−5^	*p* = 1.98 × 10^−6^	*p * = 7.47 × 10^−3^	*p* = 0.154
	CD155 (PVR)	R = 0.029	R = 0.436	R = 0.098	R = 0.011	R = 0.227	R = 0.263	R = 0.125	R = 0.148
		*p =* 0.678	*p* = 2.41 × 10^−11^	*p* = 0.151	*p* = 0.874	*p* = 8.28 × 10^−4^	*p* = 1.00 × 10^−4^	*p* = 0.067	*p* = 0.030
	B7-H4 (VTCN1)	R = −0.003	R = −0.059	R = −0.016	R = −0.048	R = −0.031	R = 0.034	R = 0.062	R = −0.086
		*p* = 0.963	*p* = 0.388	*p* = 0.814	*p* = 0.482	*p* = 0.657	*p* = 0.624	*p* = 0.369	*p* = 0.210

Bioinformatics analysis of TCGA data of 214 melanoma specimens provided by the R2 database (https://hgserver1.amc.nl/ (accessed on 12 February 2021)) was performed. Statistically significant positive (red) or negative (green) correlations (*p* < 0.05) are highlighted. Background color: One group of factors belong to the brighter grey. The 2nd group to the darker grey.

**Table 5:** Correlation of miR expression with enzymes known to be involved in 2′-O-methylation or pseudouridylation of miRs.

Induced miRs in MM with Diagnostic/Prognostic Relevance	So Far in Literature Mentioned Enzymes with Putative Role for 2′-O-Methylation of miRs		So Far in Literature Mentioned Enzymes with Putative Role for Pseudouridylation of miRs	
	FBL	HENMT1	DKC1	TRUB1
miR-9-5p	R = 0.007	R = 0.050	R = −0.026	R = 0.132
	*p* = 0.916	*p* = 0.469	*p* = 0.708	*p* = 0.053
miR-10a	R = 0.020	R = −0.036	R = −0.004	R = −0.047
	*p* = 0.767	*p* = 0.603	*p* = 0.957	*p* = 0.492
miR-10b	R = −0.012	R = 0.009	R = −0.064	R = −0.200
	*p* = 0.857	*p* = 0.896	*p* = 0.351	*p* = 3.33 × 10^−3^
miR-17-5p	n.d.	n.d.	n.d.	n.d.
miR-18a	n.d.	n.d.	n.d.	n.d.
miR-21	R = −0.168	R = 0.017	R = −0.304	R = −0.139
	*p* = 0.014	*p* = 0.800	*p* = 5.88 × 10^−6^	*p* = 0.042
miR-26b	R = 0.191	R = −0.008	R = 0.112	R = −0.091
	*p* = 4.96 × 10^−3^	*p* = 0.906	*p* = 0.101	*p* = 0.184
miR-92a	R = 0.106	R = −0.055	R = 0.155	R = 0.059
	*p* = 0.122	*p* = 0.424	*p* = 0.023	*p* = 0.390
miR-221	R = −0.029	R = 0.100	R = 0.016	R = 0.064
	*p* = 0.676	*p* = 0.144	*p* = 0.815	*p* = 0.352
miR-222	R = −0.015	R = 0.083	R = 0.083	R = −0.073
	*p* = 0.825	*p* = 0.227	*p* = 0.224	*p* = 0.285
miR-126	R = 0.088	R = −0.025	R = −0.034	R = −0.091
	*p* = 0.198	*p* = 0.711	*p* = 0.620	*p* = 0.187
miR-145	n.d.	n.d.	n.d.	n.d.
miR146a	R = −0.015	R = −0.081	R = 0.003	R = 0.098
	*p* = 0.822	*p* = 0.235	*p* = 0.968	*p* = 0.154
miR-182	R = 0.008	R = −0.068	R = −0.045	R = 0.071
	*p* = 0.905	*p* = 0.322	*p* = 0.510	*p* = 0.299
miR-514	R = −0.110	R = 0.065	R = 0.011	R = 0.052
	*p* = 0.109	*p* = 0.340	*p* = 0.874	*p* = 0.446
miR-520d	n.d.	n.d.	n.d.	n.d.
miR-527	n.d.	n.d.	n.d.	n.d.

Statistically significant positive or negative correlations (*p* < 0.05) are highlighted in red or green. Background color: One group of factors belong to the brighter grey. The 2nd group to the darker grey.

## Data Availability

The data presented in this study are available on request from the corresponding author.

## Abbreviations

APM, antigen processing machinery; BCL2, BCL2 apoptosis regulator; CCNA1, cyclin A1; CD44, cluster of differentiation 44; CENPF, centromere protein F; EMT, epithelial to mesenchymal transition; GAR1, GAR1 ribonucleoprotein; HLA, human leukocyte antigen; lncRNA, long non-coding RNA; MAPK, mitogen-activated protein kinase; MART-1, Melanoma Antigen Recognized by T-cells; MCM2, minichromosome maintenance complex component 2; MCM4, minichromosome maintenance complex component 4; MHC, major histocompatibility complex; MKI67, marker of proliferation Ki-67; MITF, melanocyte inducing transcription factor; MM, malignant melanoma; MMP2, matrix metallopeptidase 2; miR, microRNA; mRNA, messenger RNA; n.d., not determined; NHP2, NHP2 ribonucleoprotein; NM23, NME/NM23 nucleoside diphosphate kinase 1; NOP, nucleolar protein; PCNA, proliferating cell nuclear antigen; PMEL; premelanosome protein; PUS1, pseudouridine synthase 1; PUS7L, pseudouridine synthase 7 like; RBP, RNA-binding protein; RPUSD3, RNA pseudouridine synthase D3; rRNA, ribosomal RNA; S, Svedberg; S100B, S100 calcium binding protein B; scaRNA, small cajal body RNA; sncRNA, small non-coding RNA; snRNA, small nuclear RNA; SNU13, small nuclear ribonucleoprotein 13; TCGA, The Cancer Genome Atlas; TE, translational efficiency; tRNA, transfer RNA; UV, ultraviolet.
